# Printing bone in a gel: using nanocomposite bioink to print functionalised bone scaffolds

**DOI:** 10.1016/j.mtbio.2019.100028

**Published:** 2019-09-16

**Authors:** G. Cidonio, M. Cooke, M. Glinka, J.I. Dawson, L. Grover, R.O.C. Oreffo

**Affiliations:** a Bone and Joint Research Group, Centre for Human Development, Stem Cells and Regeneration, Institute of Developmental Sciences, Faculty of Medicine, University of Southampton, Southampton, SO16 6YD, UK; b School of Chemical Engineering, University of Birmingham, Edgbaston, B15 2TT, UK; c Institute of Inflammation and Ageing, MRC Musculoskeletal Ageing Centre, Queen Elizabeth Hospital Birmingham, Edgbaston, B15 2WB, UK

**Keywords:** Laponite®, Gellan, Bioinks, Biofabrication, Growth factor delivery, Skeletal tissue

## Abstract

Free-form printing offers a novel biofabrication approach to generate complex shapes by depositing hydrogel materials within a temporary supportive environment. However, printed hydrogels typically lack the requisite mechanical properties and functionality of the desired tissue, limiting application and, more importantly, safety and efficacy of the implant. The study authors have developed an innovative nanoclay-based bioink to print high shape fidelity functional constructs for potential skeletal application. Laponite® (LAP) nanoclay was combined with gellan gum (GG) to generate a printable hydrogel that was highly stable *in vitro,* displayed limited swelling ability compared with the silicate-free control and remained stable over time. An agarose fluid gel was found to provide the requisite support for the deposition of the material ink and preservation of the printed structure before crosslinking. Printed C2C12 myoblasts remained viable and displayed extensive proliferation over 21 days in culture. Cell-laden scaffolds demonstrated functionality within 1 day of culture *in vitro* and that was preserved over 3 weeks. Analysis of absorption and release mechanisms from LAP-GG using model proteins (lysozyme and bovine serum albumin) demonstrated the retention capability of the clay-based materials for compound localisation and absence of burst release. Vascular endothelial growth factor ​was loaded within the agarose fluid gel and absorbed by the material ink via absorption during deposition. The 3D-printed constructs were implanted on the chorioallantoic membrane of a 10-day-old developing chick. Extensive and preferential vasculature infiltration was observed in LAP-GG–loaded vascular endothelial growth factor constructs compared with controls (*p*<0.01 and *p*<0.0001) after only 7 days of incubation. The current studies demonstrate, for the first time, the application of innovative LAP-GG 3D constructs in the generation of growth factor–loaded 3D constructs for potential application in skeletal tissue repair.

## Graphical abstract

*LAP-GG printing in agarose fluid gel for skeletal tissue engineering.* Extrusion of LAP-GG in an agarose bed to generate three-dimensional structures (a). Low polymeric content nanosilicate bioink was used to deliver cells and preserve their viability after extrusion. Agarose fluid gel was harnessed to enable loading of compounds of interests during printing (b) in LAP-GG and GG controls. LAP-GG bioink offers the potential for enhanced compound penetration within the printed strand. LAP-GG extrusion within self-healing agarose can be used to fabricate large functional structures (c) such as 3D sleeve (i and ii) and 3D lattice (iii and iv) structures to fill critical bone defects. Cells were printed in LAP-GG (d) and viability (i and ii) and functionality (iii and iv) were assessed for skeletal tissue formation potential. Integration with chick membrane model and vascular penetration (e) of printed scaffolds implanted within the vascularised membrane of a developing chick. Scale bar: (a) 5 ​mm, (b—i and ii) 100 ​μm, (c—i and ii) 30 ​mm, (c—iii) 30 ​mm, (c—iv) 10 ​mm, (d—i) 100 ​μm, (d—iii and iv) 2 ​mm.

**Figure:**
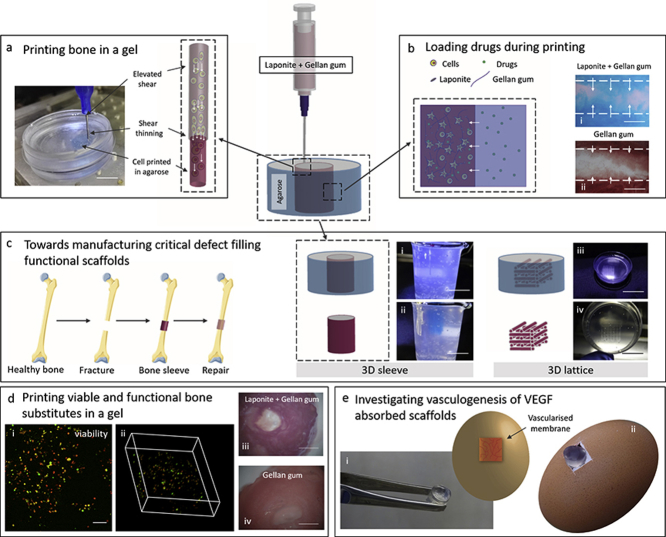

## 1 Introduction

The printing of cells in three dimensions (3D) for the fabrication of large structures targeted at critical-sized bone tissue defects has proved challenging [1]. In recent years, hydrogels have been presented as attractive biomaterials for the printing of cells (bioinks) and the generation of 3D structures [[2], [3], [4]]. A printed scaffold should, ideally, offer significant functional capacity after printing. Functionality can depend on: i) the inherent bioactivity of the hydrogel, ii) inclusion of cells and iii) retention of growth factors. Thus, a prerequisite is the potential of a hydrogel to be extruded with high precision, enabling the fabrication of complex 3D structures that retain their shape in culture. Although hydrogel formulations typically offer excellent swelling capacity, as a consequence of their ability to absorb water and significantly increase their volume [5]; for bioprinting applications, material inks should display minimal swelling capacity after printing and preserve their shape indefinitely, unless undergoing degradation. A key challenge is the fabrication of clinical-size constructs via layer-by-layer deposition of soft hydrogel in 3D. Recently, the deposition of bioinks within a temporary support material ​has demonstrated the ability to generate intricate-shaped constructs, at scale, to date impossible to produce with standard 3D printing technology [[6], [7], [8], [9]]. This novel platform, free-form printing, enables the generation of free-floating fibres in a support gel bath with the capacity for additional processing and removal by crosslinking [6,7] or reverse embedding [8,9]. Hinton et al. [10] demonstrated that the extrusion of the hydrophobic polydimethylsiloxane ​in a hydrophilic Carbopol (poly [acrylic acid]) bed resulted in high shape fidelity constructs, with the possibility to fabricate hollow and complex vasculature structures. However, the lack of further study of polydimethylsiloxane biocompatibility and Carbopol bed functionality, has limited free-form printing application using this approach. Similarly, Jin et al. [7] engineered a printing process depositing a series of hydrogels (e.g. alginate, gelatin and SU-8) in a nanoclay supporting bath. Laponite® (LAP) is a smectite clay of 1 ​nm in thickness and 25–30 ​nm in diameter holds a large surface area with a negative charge face and weak positive rim charge [[11], [12], [13]]. In an aqueous environment, LAP nanodiscs self-assemble in a “house-of-cards” structure, resulting in an interconnected network. The use of LAP as a supporting bath allowed the fabrication of complex structures; however, extrusion of material with pH <7 was limited leading to nozzle clogging and the need for special chemical treatment [7].

Recently, Moxon et al. [6] detailed the ability to extrude a free-form bioink into a cytocompatible gel bath enabling the support and further crosslinking of the bioink. The hydrogel ink of choice was gellan gum (GG) [14,15], a polysaccharide material that was biocompatible [16], supporting cell growth and functionality *in vitro* [17]. GG is a Food and Drug Administration and European Union (E418) approved biomaterial of microbial origin (from *Sphingomonas elodea*) with advantageous physicochemical properties, widely used as an additive for pharmaceutical products [18] and in tissue engineering applications [19]. In the work by Moxon et al. [6]*,* an agarose (a widely used polymeric biomaterial that can form an inert hydrogel network widely used for cell encapsulation [20,21]) fluid gel support bath was used for printing. The fluid gel was able, at the same time, to support the extruded GG bioink before crosslinking and allow the free movement of the printing nozzle by self-healing immediately after printing. GG at low concentration could be precisely deposited in a 3D format, generating complex structures. A complex double-layered osteochondral plug was generated by depositing a GG solution loaded with hydroxyapatite ​to mimic bone and GG as the cartilage mimicking layer. The resulting construct was functional, although lacked shape complexity, a defined drug-eluting mechanism and only *in vitro* investigations were performed*.*

GG is an attractive biomaterial to study given the potential to tune the mechanical properties of the material by modulation of the system temperature [22]. This particular property is attractive given the opportunity to modulate the chemical and physical modification of this polymeric material [23]. LAP has been shown to be an excellent filler for hydrogels targeted as bioinks for biofabrication applications [12,24,25]. Pacelli et al. [23] recently reported the physicochemical and drug-delivery properties of LAP-GG hydrogels, demonstrating that: i) LAP induced an increase in viscosity of the final polymeric solution, ii) stabilised the GG network and iii) modulated the release of a model drug (ofloxacin). However, functional studies on encapsulated cells were not investigated and the use of a methacrylate-modified GG, crosslinked using UV-light provides a challenging strategy for viable cell encapsulation purposes.

In the present study, the authors have developed a nanocomposite bioink (LAP-GG) that can be extruded in a tailored functional agarose fluid gel bed. Specifically, this study has examined the potential to increase the printing resolution of GG hydrogel after LAP inclusion, fabricating a novel nanocomposite. In addition, the present study has determined the potential of the biomaterial composite to deliver viable cells in 3D and to sustain proliferation and functionality within an *ex vivo* ​chick chorioallantoic membrane (CAM) model to investigate vasculature integration and tissue formation.

## 2 Materials and methods

### 2.1 Biomaterials

#### 2.1.1 Laponite®

After sterilising LAP ​(Na^+^_0.7_ [(Mg_5.5_Li_0.3_) Si_8_O_20_^−^ (OH)_4_]^−^_0.7_ XLG grade, BYK Additives & Instruments, UK) by an autoclaved cycle (126 ​°C, 1.4 ​bar), the powder was poured slowly into a beaker with sterile deionised water (DW) and stirred for at least 6 ​h with a magnetic stirrer in a class II sterile hood. Addition of LAP powder to DW was carried out serially, adding a small portion of LAP and leaving the suspension to stir at least for 3 hours, allowing full dispersion of nanodiscs confirmed by the increase in optical transparency.

#### 2.1.2 Gellan gum

Low acyl GG (Gellan Gum Low Acyl [Type F, USP grade], Azelis, UK) was allowed to dissolve in DW at different concentrations, as detailed previously [6]. Hydrogels were prepared based on w/v percentage formulations. DW was left to stir and heated at 60 ​°C before adding GG and leaving it to stir for 1 ​h ​at constant shear and temperature. The increase of solution transparency confirmed the GG complete dispersion. GG gel was allowed to cool down at room temperature for 2 ​h before the GG gel was used for rheological, printing or other studies.

#### 2.1.3 Laponite®-gellan gum

LAP-GG ​was prepared from an LAP suspension by a serial inclusion and direct mixing of GG. Briefly, DW was left to stir and LAP was added slowly to reach clear dispersion after 6 ​h. Sterile GG powder was then added to the LAP suspension and heated at 60 ​°C. GG integration in LAP suspension was carried out at a constant shearing at 60 ​°C for 1 ​h. Final LAP-GG suspension results homogenous and amber in colour ready for printing. If stored at room temperature, the LAP-GG bioink was observed to harden.

#### 2.1.4 Agarose fluid gel

Fluid gel was prepared as previously described [6]. Briefly, a 0.5% w/v solution of agarose (low gelling agarose, Genetic analysis grade, Fisher Bioreagents, UK) was autoclaved immediately after agarose addition to 300 ​mL of DW. The solution was allowed to cool down from 85 ​°C to 20 ​°C under constant shear using a magnetic stirrer. The solution was sterilised under UV irradiation for 1 ​h before the use as a support for printing.

### 2.2 Mass loss and swelling ratio

Mass loss and swelling studies were performed on the GG and LAP-GG discs to study the effect of nanoclay incorporation into GG hydrogels as previously detailed [26]. GG and LAP-GG disc samples (500 ​μL) were weighed immediately before ($m_{initial}$) and after crosslinking ($m_{initial,\;t0}$) to obtain initial wet mass, and three samples were lyophilized to obtain their dry weights ($m_{dry,\;t0}$). The macromer fraction was calculated using the following equation (1):

(1) $$actual\;macromer\;fraction=\frac{m_{dry,\;t0}}{m_{initial,\;t0}}$$

The remaining samples were incubated in a phosphate-buffered saline (PBS, Thermo Fisher Scientific) or Hanks' balanced salt solution (HBSS, Thermo Fisher Scientific) bath at 37 ​°C to allow swelling and the soluble fraction to leach from the hydrogel network. After 1 day, the samples were weighed again ($m_{swollen}$). The samples were then lyophilized and weighed a last time ($m_{dry}$). The sol fraction was defined as the mass loss after 1 day and was calculated using equations (2), (3). Mass swelling ratio (*q*) was calculated using equation (4).

(2) $$m_{initial,\;dry}=\text{ ​}m_{initial}\;\left(actual\;macromer\;fraction\right)$$

(3) $$sol\;fraction=\text{ ​}\frac{m_{initial.dry}-\text{ ​}m_{dry}}{m_{initial,dry}}\text{ ​}100\text{\%}$$

(4) $$q=\text{ ​}\frac{m_{swollen}}{m_{dry}}$$

### 2.3 Acellular printing of GG and LAP-GG

Printing of acellular GG and LAP-GG was carried out in air and agarose. A previously used custom-made 3D bioprinter [27] was used to generate ​3D scaffolds. A 5 ​mL agarose fluid gel bed was prepared as a supportive bath for extrusion. LAP-GG and GG were labelled with visible colour dyes (Alcian Blue and Sirius Red) to enable visualisation during extrusion in the agarose fluid gel bed. A hollow cylindrical shape of 5 ​mm in diameter was used as blueprint. Printing was carried out at a flow rate of 2.5 ​mm/s^-^ ​depositing 10 layers with an average layer height set to 300 ​μm distance. A 1 ​M CaCl_2_ solution was used to further crosslink GG and LAP-GG for 10 ​min.

### 2.4 Swelling in air and agarose

To evaluate the swelling behaviour of the bioink after printing in agarose fluid gel, GG and LAP-GG fibres were extruded from a cylindrical blunt nozzle (inner diameter: 250 ​μm). Strands were deposited in a 2 ​mm thick agarose fluid gel. Measurements were carried out on GG (n ​= ​3) and LAP-GG (n ​= ​3) strands imaged using a stereo light microscope immediately after printing and after crosslinking with 1 ​M CaCl_2_ for 10 ​min. Additional measurements were acquired 1, 3 and 24 ​h after printing similarly to the authors' previous study [23]. To evaluate whole scaffolds swelling, a 5 ​mm hollow cylindrical structure was printed in agarose and air. Measurements of the internal (pore) and outer diameter were carried out on images acquired using a stereo light microscope. All measurements were analysed with ROI macro plugin in Image J (1.44p, National Institutes of Health, Bethesda, Maryland, USA), which was used as the analysing software.

### 2.5 C2C12 ​cell culture

The immortalized mouse premyoblast cell line C2C12 (mouse C3H, muscle) was obtained from the European Collection of Authenticated Cell Cultures, Public Health England, Porton Down, Salisbury, SP4 0JG, UK. The cells were maintained in culture supplemented with full cell culture media (Dulbecco's Modified Eagle Medium [DMEM] supplemented with 10% (v/v) fetal bovine serum (FBS), 1% (v/v) penicillin/streptomycin (Pen/Strep), replenished every 3 days and cells passaged at a maximum confluence of 80–90%.

### 2.6 Microscopy of cast gels and cell attachment

0.1 ​× ​10^6^ ​cell C2C12 ​cells were seeded onto 100 ​μL LAP-GG and GG gels plated in wells of a 96-well plate. Cells were cultured for 24 ​h to allow attachment with full media. Scanning electron microscopy (SEM, FEI Quanta 250 FEG, operated in SEM mode) at a voltage of 5 ​kV (spot size 3) was used to image acellular and cellular gels. Before SEM, samples were dehydrated with a freeze-drier (Lablyo Mini, Froze in Time Ltd., UK) for 12 ​h and coated with platinum (Q150TES, sputter coater, UK). Energy-dispersive x-ray spectroscopy analysis was performed at a voltage of 12 ​kV after carbon coating.

### 2.7 Rheology

Rheological characterisation of LAP-GG (1% w/v LAP and 2% w/v GG) composite was carried out using a rotary rheometer (Anton Parr, MCR92). The planar plate geometry (PP25) was used for all the rheological tests. A range of shear rate between 0.1 and 100 per s^-^ was applied to measure viscosity (Pa s) and shear stress (Pa). Amplitude sweep analysis was carried out between 0.1 and 100% of strain. Frequency sweeps of 26 steps were carried out by maintaining a 1% strain over a range from 0 to 100 ​Hz, finding storage and loss moduli (Pa), respectively.

### 2.8 C2C12 ​cell printing in agarose fluid gel

Cell-laden scaffolds were fabricated with GG and LAP-GG hydrogel. C2C12 ​cells were expanded for 14 days. A total of 5 ​× ​10^6^ ​cell/mL of materials were prelabelled with protocol previously reported [12]. Briefly, cells were suspended at a density of 1 ​× ​10^6^ ​cells/ml ​in serum-free culture medium and Vybrant® DiD (Cell-Labeling Solution, V-22887, Molecular Probes) was added following the manufacturing protocol. The cell suspension was incubated for 20 ​min ​at 37 ​°C, 5% CO_2_. The cell suspension was subsequently centrifuged and the supernatant was removed. Washing with serum-free media was carried out to remove any unbound DiD. Cells were then encapsulated in GG and LAP-GG and printed. Printing was carried out in agarose fluid gel. The extrusion of hollow cylinders (5 ​mm in diameter and 10 layers high) were undertaken at a constant printing speed of 2.5 ​mm/s. Printed scaffolds were crosslinked for 10 ​min using 1 ​M CaCl_2_, subsequently washed with HBSS ​to remove excess agarose gel [6] and incubated in full cell culture media at 37 ​°C and 5% CO_2_ balanced air up to 21 days. Scaffolds printed using the two bioinks were divided into two additional groups to obtain scaffolds for cell viability (n ​= ​9 cell-laden and n ​= ​9 acellular) and functionality (n ​= ​12 cell-laden and n ​= ​12 acellular) investigations.

### 2.9 Viability of cell-laden printed scaffolds

Viability at day 1, 7 and 21 was investigated using Calcein AM staining and confocal imaging as previously described [12]. Briefly, samples were washed twice with 1 ​× ​HBSS. Calcein AM (C3099, Invitrogen, Thermo Fisher Scientific) was diluted in serum-free culture media and added to each cell-laden scaffold. The scaffolds were incubated at 37 ​°C and 5% CO_2_ balanced air for 1 ​h, following the manufacturer's protocol. A repeated wash with 1 ​× ​HBSS was subsequently carried out. Samples were imaged using a confocal scanning microscope (Leica TCS SP5, Leica Microsystems, Wetzlar, Germany). Living cells were identified when stained with Calcein AM and DiD. Metabolically inactive (dead) cells were identified by DiD staining. Cell viability was quantified by comparing the number of viable and dead cells in the Z-stacks of 15 images per time point (n ​= ​3) as previously reported [12]. Analysis was carried out using Image J “Analyze Particle” tool with a threshold range of 20–600 pixel to avoid background interference from LAP autofluorescence. Cell density was calculated by normalisation of viable cells and the volume of interests. The percentage values were plotted with reference to the day 1 group established as 100%.

### 2.10 Functionality of cell-laden printed scaffolds

C2C12-laden 3D printed scaffolds were conditioned with basal (DMEM supplemented with 10% [v/v] FBS, 1% [v/v] Pen/Strep) or osteogenic (DMEM supplemented with 10% [v/v] FBS, 1% [v/v] Pen/Strep, 100 ​μM ascorbate-2-phosphate, 10 ​nM dexamethasone ​and 10 ​nM vitamin D [1α,25-OH_2_-Vit D3]) media and cultured up to 21 days. Samples were fixed in 95% ethanol for 10 ​min. The ethanol solution was removed, and samples were washed twice with 1 ​× ​HBSS. Alkaline phosphatase (ALP) staining solution was prepared from 9.6 ​ml of DW, 400 ​μL Naphtol (AS-MX Phosphate Alkaline Solution, 85-5, Sigma, UK) and Fast Violet Salt (F1631 Sigma, UK). The solution was added to the samples, which were incubated at 37 ​°C for 1 ​h. The reaction was then stopped by 1 ​× ​HBSS. Samples were stored at 4 ​°C overnight and imaged with Zeiss Axiovert 200 (Carl Zeiss, Germany) the following day. Mean intensity units of each image were calculated using Image J software, by analysing region of interest (ROI) with the “density” tool present in Fiji (1.44p, National Institutes of Health, Bethesda, Maryland, USA).

### 2.11 Drug analogues absorption and release

Bovine serum albumin (BSA, Sigma-Aldrich) and lysozyme (lysozyme from chicken egg white, lyophilized powder, protein ≥90%, ≥40,000 units/mg protein, Sigma-Aldrich) were solubilised in HBSS at 100 ​and 10 ​μg/mL, respectively. Before absorption, GG and LAP-GG were cast to generate cylindrical scaffolds. Scaffolds (n ​= ​3) were soaked in BSA or lysozyme solutions for 1, 2, 4, 8 and 24 ​h and the supernatant subsequently collected. After 24 ​h, BSA and lysozyme solutions were replaced with collagenase D (from *Clostridium histolyticum*, Roche Diagnostics GmbH). Blot dry sample weight was registered before and after collagenase addition. A reduction in weight from 161.5 ​± ​4.3 ​to 97.6 ​± ​3.2 ​μg was noted. Samples were collected (n ​= ​3) and readings determined at 1, 2, 4, 8 and 24 ​h after collagenase addition. Absorbed and released concentration values were quantified using a microplate reader (GloMax Discover, Promega) using a protein quantification kit (Protein quantification kit—Rapid, Sigma-Aldrich, UK) according to the manufacturer's protocol.

To investigate the possibility to solubilise drugs in the agarose fluid gel and induce the absorption of the drugs in the bioink during and after printing, a cationic dye of different colours was used to tag the agarose gel and before printing of either GG or LAP-GG. Fibres extruded from a 250 ​μm nozzle were deposited in coloured agarose. A stereo and an inverted (Zeiss Axiovert 200) microscope were used to image the printed structures and evaluate penetration by sectioning of the strands at the extremity and in a median portion of the construct.

### 2.12 CAM assay

Before implantation, scaffolds were printed using sterile LAP-GG and GG bioinks. Recombinant human vascular endothelial growth factor (VEGF 165, PeproTech, USA) was solubilised at 100 ​μg/mL ​in a solution of 0.5% w/v agarose fluid gel. Printing was performed by depositing a 20-layer high bulk cylinder of 10 ​mm in diameter into VEGF-absorbed agarose support bath. A total of n ​= ​8 scaffolds were fabricated with either LAP-GG or GG alone for VEGF absorption. VEGF was absorbed at 100 ​μg/mL^-^ ​onto 3D-casted discs. A remaining n ​= ​8 constructs were printed with either LAP-GG or GG alone in VEGF-free agarose. Crosslinking of scaffolds (10 ​min exposure to 1 ​M CaCl_2_ solution) was carried out after 15 ​min. Both printed GG and LAP-GG cylinders were maintained in agarose loaded with VEGF for 30 ​min before collection and a single wash with HBSS before implantation on the CAM.

Animal procedures were undertaken in accordance with the guidelines and regulations of the Animals Act 1986, UK. The chick CAM ​model assay was conducted under Home Office Approval UK (Project licence—PPL P3E01C456). Chicken eggs were incubated in a Hatchmaster incubator (Brinsea, UK) for 10 days at 37 ​°C in a 60% humidified atmosphere and 1 ​h rotation. After 10 days of fertilisation, a 2 ​cm^2^ window was created on the eggshell using a scalpel blade. Individual 3D scaffolds (n ​= ​10) were inserted on the CAM. The eggs were sealed with sterile parafilm and incubated without rotation. Chick embryos were inspected daily for growth and viability by candling. After 7 days of incubation, 3D samples were harvested and CAM integration inspected using a stereomicroscope fitted with a digital camera (Canon Powershot G2). Chalkley score (overlap morphometry) was carried out as previously described [28]. Briefly, integrated samples were assessed by matching the Chalkley graticule and the vasculature of the CAM in correspondence of the implant area. The score was quantified by counting the collision points of the dotted graticule and the vasculature in proximity of the implanted scaffolds.

### 2.13 Statistical analysis

GraphPad Prism 7 (GraphPad Software Inc., La Jolla, CA) was used for statistical analysis. D'Agostino--Pearson normality test was used to assess the differences in the data. Non-significant differences were defined as *p*>0.05. When multiple comparison tests were performed for the data analysed using one-way and two-way analysis of variance, a correction using Sidak or Tukey testing was carried out.

## 3 Results

### 3.1 Physical characterisation of LAP-GG nanocomposite

**Fig. 1:**
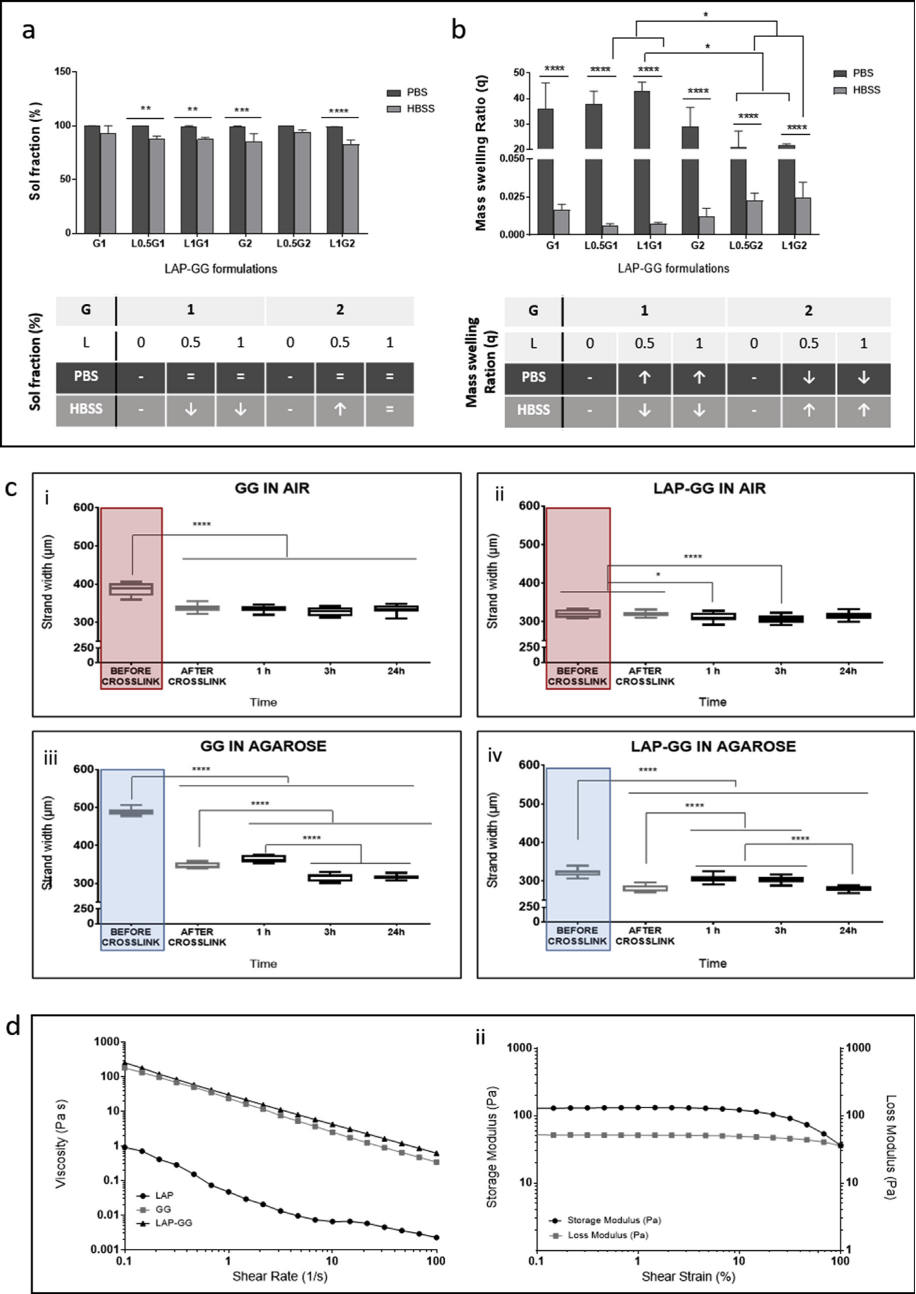
Swelling and rheological properties of nanosilicate composite gels. (a) Sol fraction ​of LAP-GG formulations and controls evaluated in PBS or in HBSS. (b) Mass swelling ratio of LAP-GG gels compositions and controls in PBS and HBSS. (c) Measurements were collected for GG (c—i) and LAP-GG (c—ii) printed in air. Fibre size for printed GG (c—iii) and LAP-GG (c—iv) printed in agarose. Viscosity (d—i) drop by the change of shear rate was registered for LAP, GG and LAP-GG. Storage and loss moduli (d—ii) of LAP-GG bioink was measured depending on the percentage change in shear strain. Statistical significance was assessed by two-way analysis of variance. Mean ​± ​S.D. (a, b) n ​= ​6, (c) n ​= ​3; *p<0.05, **p<0.01, ***p<0.001, ****p<0.0001. LAP ​was labelled as L and GG as G, with the subsequent number indicating the % w/v. GG, gellan gum; HBSS, Hanks' balanced salt solution; LAP, Laponite®; PBS, phosphate-buffered saline.

To determine the ability of the LAP-GG nanosilicate-modified hydrogels to withstand swelling forces, the physical properties of cast gels were investigated for sol fraction content (Fig. 1a) and mass swelling ratio (Fig. 1b). PBS and HBSS were used to test the LAP-GG physical characteristics depending on the ionic content. A series of formulations were tested by varying GG between 1% and 2% w/v, and LAP between 0.5% and 1% w/v.

The sol fractions of GG and LAP-GG samples immersed in PBS and HBSS were not significantly different (Fig. 1a). However, a significant increase in sol fraction was observed when L0.5G1 (*p*<0.01), L1G1 (*p*<0.01), G2 (*p*<0.001), and L1G2 (*p*<0.0001) were immersed in PBS compared with HBSS. The mass swelling ratio (Fig. 1b) in PBS was found to increase with the addition of LAP to GG. In particular, L1G1 displayed a significantly greater swelling percentage (*p*<0.05) compared with L0.5G2 and L1G2. Assessment of swelling ratio in HBSS showed significantly reduced swelling compared with PBS conditioning, for all formulations (*p*<0.0001). LAP inclusion induced a decrease in swelling ratio, whereas the addition of GG was reported to significantly (*p*<0.05) increase the swelling ratio between L0.5G1-L1G1 and L0.5G2-L1G2 formulations.

**Supporting Fig. 1:**
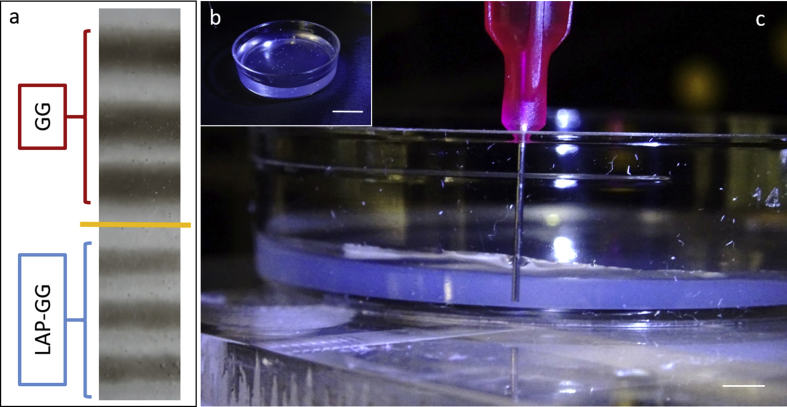
Printing stability and swelling kinetics in agarose. (a) GG and LAP-GG stands printed in agarose fluid gel. (b) Agarose gel loaded at 2 ​mm in thickness. (c) Printing of strand for swelling test. Scale bar: (b) 10 ​mm, (c) 2 ​mm.

To validate the sol fraction, mass swelling studies and to investigate the bioink print fidelity, GG and LAP-GG were printed in air (Fig. 1c—i and ii) or in agarose gel (Fig. 1c—iii and iv) and strands measured for changes in swelling over 24 ​h. A bioink formulation of 2% w/v content in GG and 1% LAP was selected for all further characterisation studies. GG and LAP-GG fibres (Supporting Fig. 1a) were printed in an agarose bed (Supporting Fig. 1b) with a cylindrical nozzle of 250 ​μm (I.D.) (Supporting Fig. 1c) to generate a 250 ​μm deposited strand. GG printing in air (Fig. 1c—i) resulted in a swollen fibre before crosslinking, which was significantly larger (*p*<0.0001) than immediately after crosslinking, or after 1, 3 and 24 ​h. LAP-GG, when printed in air, (Fig. 1c—ii), displayed a lower degree of swelling compared with GG and a comparable dimension of strand size immediately after crosslinking. A significant decrease in strand diameter was registered after 1 (*p*<0.05), 3 ​h and 24 ​h (*p*<0.0001). The use of an agarose gel bed significantly reduced both swelling and strand diameter of printed GG (Fig. 1c—iii). After CaCl_2_ crosslinking, the strand was smaller (*p*<0.0001) in diameter compared with the printed strand before crosslinking.

**Supporting Fig. 2:**
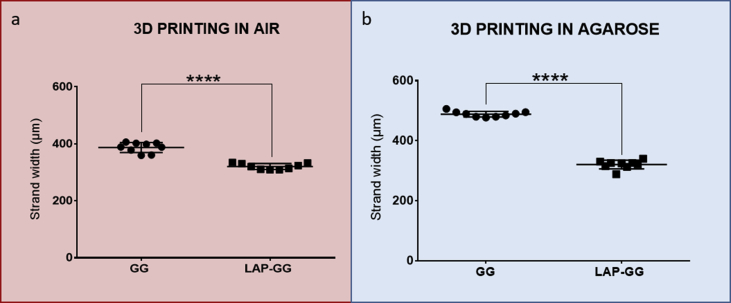
Comparison of fibre diameter immediately after deposition. Measurements were carried out on samples printed both in air (a) and in agarose (b). Statistical significance was assessed by two-way ANOVA. Mean ​± ​S.D. n ​= ​3 *p<0.05, ****p<0.0001.

However, after only 1 ​h, the diameter had significantly swollen compared with strands before and after crosslinking (*p*<0.0001), after 3 and 24 ​h (*p*<0.0001). LAP-GG (Fig. 1c—iv) generated a significantly lower degree of swelling immediately before crosslinking (*p*<0.0001) compared with GG bioink. LAP-GG displayed a significantly decreased fibre diameter (*p*<0.0001) after crosslinking. A significant swelling after 1 ​h was found, consistent with GG swelling kinetics. After 3 ​h, the strand diameter was unchanged and subsequently decreased after 24 ​h. Interestingly, the LAP-GG printed fibres before crosslinking were significantly smaller (*p*<0.0001) in diameter compared with GG, both in air (Supporting Fig. 2a) and in agarose (Supporting Fig. 2b).

The rheological properties of LAP-GG bioink were investigated before extrusion and viscosity measurements (Fig. 1d—i) were determined to evaluate bioink printability. Single components and final LAP-GG blends were tested for viscosity. LAP showed a low viscosity profile (0.92 ​Pa ​s) compared with GG (182.35 ​Pa ​s). LAP-GG components were observed to decrease linearly with an increase in shear rate (from 0.1 to 100 per s^-^). LAP-GG nanocomposites exhibited a higher viscosity (257.82 ​Pa ​s) than GG alone, despite only containing 1% w/v of LAP solid content. The reduction in LAP-GG viscosity, with increasing shear rate, followed a similar trend compared to GG. Storage and loss moduli (Fig. 1d—ii) of the LAP-GG bioink were measured according to the percentage change of the shear strain. The storage modulus was stable (131.02 ​Pa) up to 3.16% in strain, but subsequently decreased up to 36.13 ​Pa when 100% shear was reached. The loss modulus linearly decreased from 52.57 ​to 35.90 ​Pa.

### 3.2 Characterisation of printed acellular scaffolds in a fluid gel

**Fig. 2:**
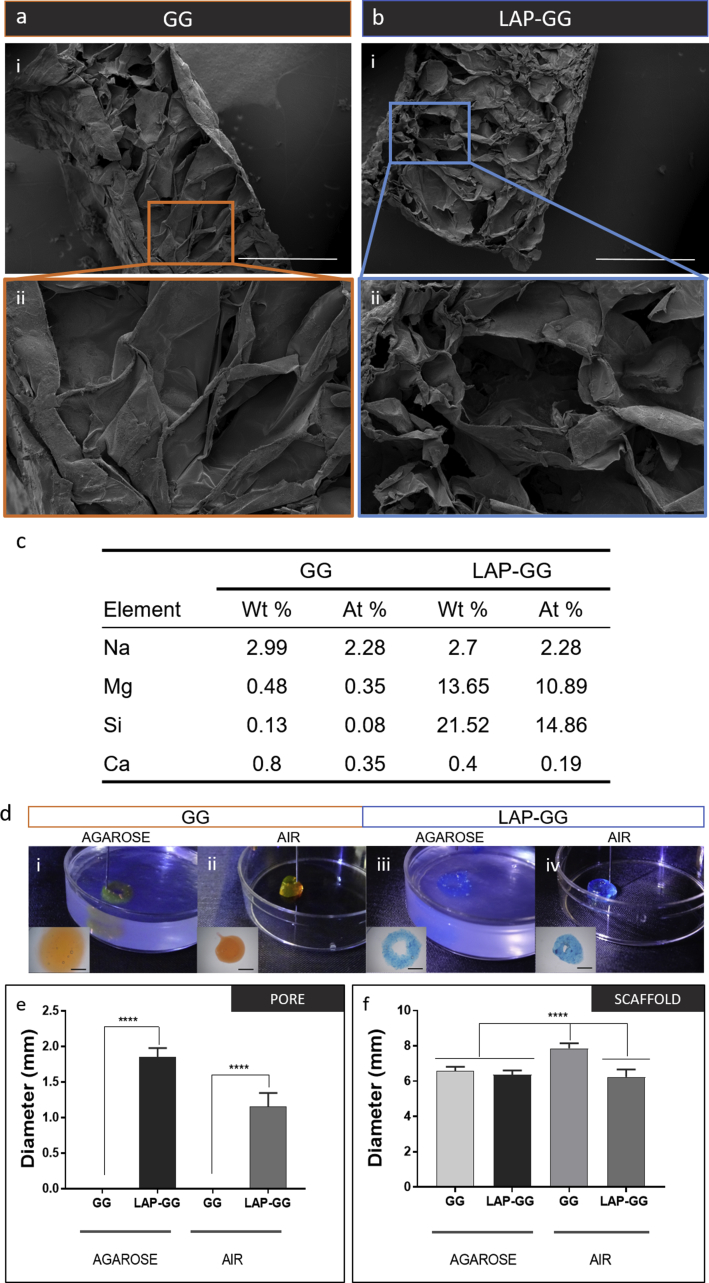
Physical characterisation of printable nanosilicate composite hydrogel. Micrographs of GG (a) and LAP-GG (b) showing internal open porosity. (c) Energy-dispersive x-ray spectroscopy (EDX) of cross section of plotted GG and LAP-GG scaffolds after element analysis. GG could be extruded in agarose gel (d—i) or air (d—ii) into circular shape. LAP-GG was deposited both in agarose (d—iii) and in air (d—iv). Measurements of the inner pore (e) and overall scaffold diameter (f) on samples printed in agarose fluid gel or air. Statistical significance was assessed by one-way analysis of variance. Mean ​± ​S.D. n ​= ​6, ****p<0.0001. Scale bar: (a—i and b—i) 1 ​mm, (a—ii and b—ii) 300 ​μm, (d—i–iv) 2 ​mm. GG, gellan gum; LAP, Laponite®.

**Supporting Fig. 3:**
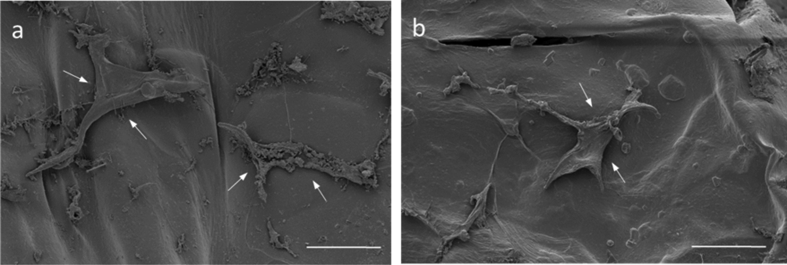
Cell attachment onto GG and LAP-GG scaffolds. C2C12 ​cells seeded on GG (e) and LAP-GG (f) scaffolds and imaged under SEM. Scale bar: (a and b) 100 ​μm.

SEM images of cast GG and LAP-GG gels (Fig. 2) were acquired and analysed. GG discs (Fig. 2a—i) displayed a less porous internal architecture compared with LAP-GG (Fig. 2b—i), evidenced at higher magnification (Fig. 2a—ii and b—ii, respectively). Elemental analysis via energy-dispersive x-ray spectroscopy (EDX) confirmed LAP inclusion and integration within the printed scaffolds (Fig. 2c). Increased percentage of magnesium (Mg) and silicon (Si) within the construct confirmed LAP inclusion. Cells seeded on GG (Supporting Fig. 3a) and LAP-GG (Supporting Fig. 3b) were observed to attach to the scaffolds and presented a similar morphology after 24 ​h of culture.

To investigate the effect of LAP addition to GG printing fidelity, GG and LAP-GG were deposited both in agarose and in air (Fig. 2d–f). Extrusion of GG in agarose medium (Fig. 2d—i) resulted in an initial formation of a cylindrical-shaped construct, which swelled immediately, until the central pore was occluded. GG extrusion in air (Fig. 2d—ii) resulted in a printed construct that collapsed immediately after a few initial layers. LAP-GG was highly printable in agarose (Fig. 2d—iii), and layer-by-layer deposition generated a high-resolution construct that closely represented the blueprint file. However, when LAP-GG was extruded in air, the structure was unstable (Fig. 2d—iv) and unable to support its own weight.

**Supporting Fig. 4:**
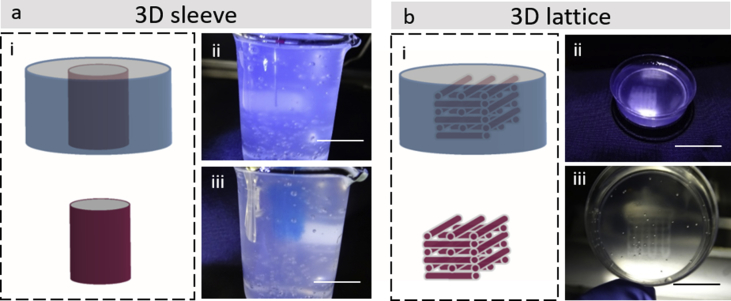
Printing 3D sleeve and lattice for repairing of large bone defects. (a) A blueprint of a 3D sleeve was generated with an initial height of 30 ​mm. Extrusion of LAP-GG (blue) in agarose fluid gel (transparent) resulted in the fabrication of a large structure (i–iii). (b) Lattice structure ​could be printed in agarose precisely resembling blue-print file (i–iii). Scale bar: (a—i and ii) 30 ​mm, (b—i and ii) 10 ​mm.

Measurements were conducted on cylindrical-shaped 3D constructs to investigate print fidelity (Fig. 2e–f). GG swelled immediately on extrusion resulting in a collapsed central pore. Extrusion of GG bioink with the addition of LAP generated cylindrical scaffolds with significantly (*p*<0.0001) improved central pore dimensions both in agarose and in air, respectively. However, when printed in air, GG displayed a significantly (*p*<0.0001) larger overall diameter compared with constructs printed with LAP-GG bioink. Larger cylindrical constructs were printed in the agarose gel using the LAP-GG bioink (Supporting Fig. 4a). As proof-of-concept, a 30 ​mm high 3D sleeve structure was printed (Supporting Fig. 4a—i–iii), showing fidelity to the blueprint and elevated printability in the agarose gel. Lattice scaffolds (Supporting Fig. 4b) were printed in agarose demonstrating enhanced printing fidelity (Supporting Fig. 4b—i–iii).

### 3.3 Cell viability, proliferation and functionality of printed C2C12 ​cells *in vitro*

**Fig. 3:**
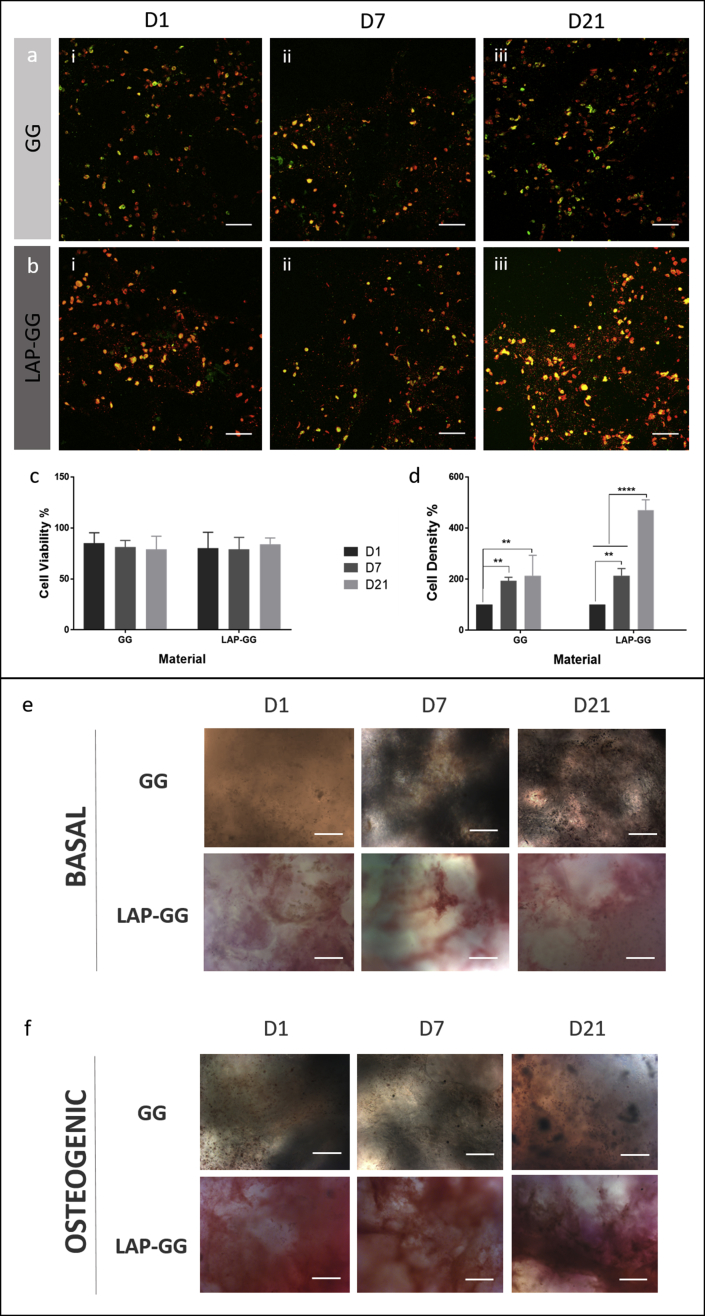
Evaluation of printed cell-laden nanocomposite bioink. Confocal microscopy images of C2C12-laden GG (a) and LAP-GG (b) scaffolds at (i) 1, (ii) 7 and (iii) 21 days. All cells were stained with DiD solution (red) with mitotically active cells stained with Calcein AM (green). Cell (c) viability and (d) density were quantified from confocal images. Cell viability percentages were obtained from the calculated difference between viable and total numbers of cells. Dead cells were assumed to comprise cells not stained with Calcein AM. Alkaline phosphatase (ALP)–stained GG and LAP-GG printed scaffolds imaged after 1, 7 and 21 days of culture in basal (e) and osteogenic (f) media. Statistical significance was assessed by two-way analysis of variance. Mean ​± ​S.D. n ​= ​3, **p<0.01, ****p<0.0001. Scale bar: (a–f) 100 ​μm. GG, gellan gum; LAP, Laponite®.

C2C12 promyoblast cells were printed in GG and LAP-GG to evaluate the functional capacity of the printed bioink *in vitro*. Cell viability (Fig. 3a and b) remained at around 80% in both treatments over the time course (Fig. 3c). Cell density (Fig. 3d) of the printed constructs was evaluated to determine the proliferative potential of the printed C2C12 ​cells in GG or in LAP-GG bioinks with the cell density after 1 day of culture set as the baseline (100%). Both GG and Lap-GG bioinks sustained a linear and significant increase in cell density over time (*p*<0.01 and *p*<0.0001 ​at day 21, respectively).

C2C12 cell-laden printed scaffolds were evaluated for their ability to host osteogenic differentiation of seeded cells over 21 days. Scaffolds fabricated with GG and LAP-GG bioink were cultured for 1, 7 and 21 days in basal (Fig. 3e) and osteogenic (Fig. 3f) media. Results demonstrated that basal conditioning of GG encapsulated C2C12 ​cells showed limited ALP staining after 1 and 7 days, with expression of ALP after 21 days *in vitro*. LAP-GG cultured in basal media showed ALP expression after 1 day of culture that displayed an increasing intensity after 7 and 21 days. Osteogenic-conditioned GG scaffolds were observed to stain for ALP after 1 day with reduced intensity after 7 days of culture *in vitro*. C2C12 cell-laden GG scaffolds demonstrated diffuse ALP expression. Noticeably darker cell agglomerates were found throughout the entire scaffold construct, suggesting mineralised nodules.

### 3.4 Drug absorption/release from composite bioink

**Fig. 4:**
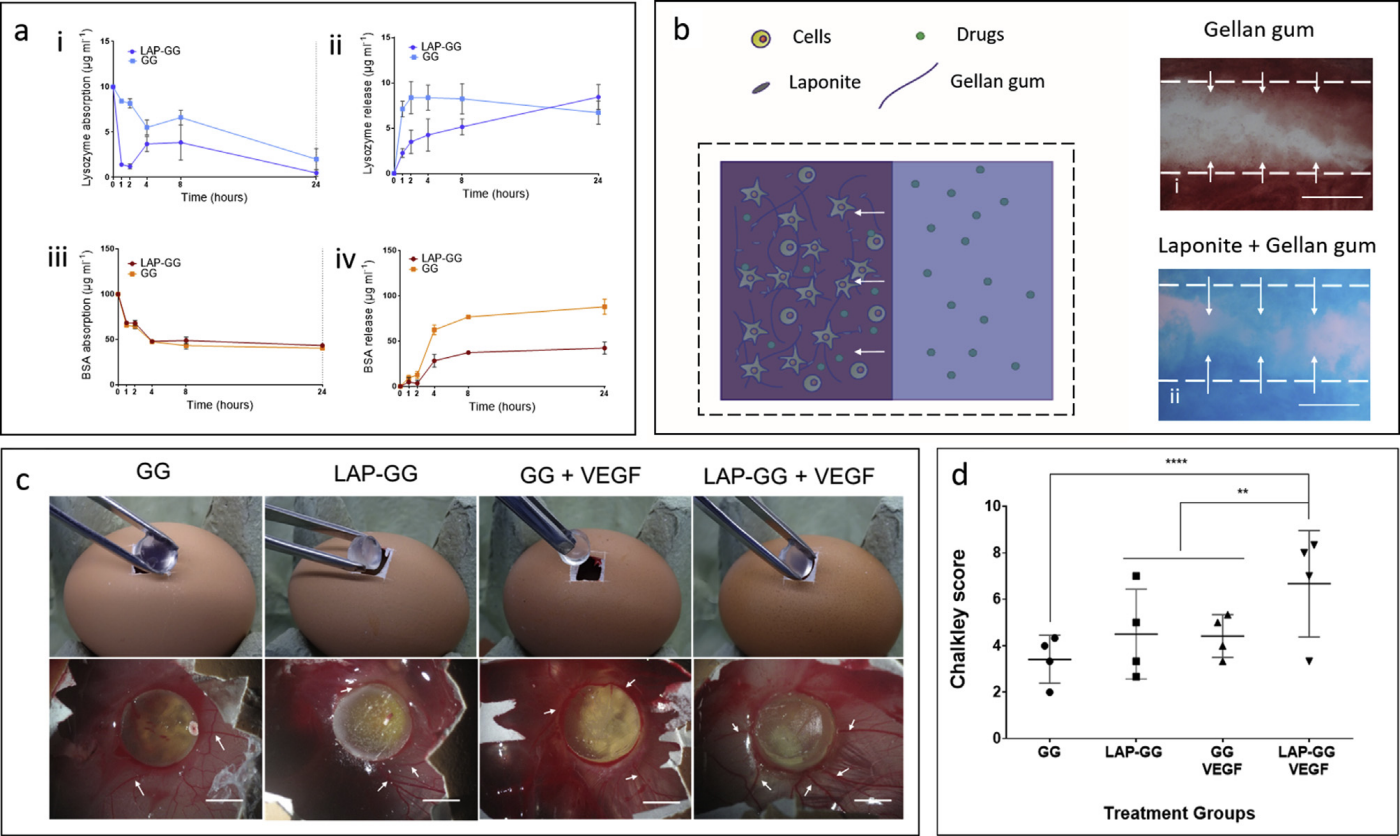
Nanoclay-based 3D scaffolds localise absorbed drugs during and after printing elicit an angiogenic response *ex vivo*. Lysozyme absorption (a—i) and release (a—ii) were performed for 24 hours, respectively. BSA absorption (a—iii) and release (a—iv) were performed for 24 hours, respectively. (b) Drugs could be solubilised in the agarose bath support and absorbed by the clay-based bioink containing viable cells post-printing. GG (b-i) and LAP-GG (b-ii) bioinks were printed in agarose tagged with red and blue dye respectively. (c) Implantation of GG, LAP-GG, GG ​+ ​VEGF and LAP-GG ​+ ​VEGF. Vascular integration assessment of GG, LAP-GG, GG ​+ ​VEGF and LAP-GG ​+ ​VEGF after 7 days of incubation. Arrows indicated major afferent vessels to the implanted scaffold. (d) Chalkley score of the vascular penetration of implanted GG and LAP-GG, drug-free or absorbed VEGF groups. Statistical significance assessed by two-way analysis of variance. Mean ​± ​S.D. n ​= ​4 **p<0.01, ****p<0.0001. Scale bar: (b-i) 100 ​μm, (b-ii, iii) 200 ​μm, (c) 5 ​mm. BSA, bovine serum albumin; GG, gellan gum; LAP, Laponite®; VEGF, vascular endothelial growth factor.

The potential for localisation and retention of drugs was tested on GG and LAP-GG constructs *in vitro*. BMP-2 and VEGF analogues (lysozyme and BSA, respectively) were used to model the load and release profiles of functional osteoinductive and angiogenic compounds, respectively. During absorption, cast gels were immersed in HBSS-based drug solution and release was later induced by collagenase digestion (Fig. 4a).

Lysozyme solution (10 ​μg/mL) was incubated with the gels over 24 ​h. The solution concentration was subsequently measured as a surrogate marker of gel absorption. GG cast gels displayed a slow absorption profile (Fig. 4a—i), whereas LAP-GG was noted to be significantly faster after only 1 ​h, similarly resuming the absorption up to 24 ​h. After 24 ​h, lysozyme solution was replaced with collagenase and lysozyme release was measured. GG (Fig. 4a—ii) released the majority of the absorbed lysozyme after 1 ​h, whereas LAP-GG showed a sustained release profile over 24 ​h.

BSA was absorbed onto GG and LAP-GG at an initial concentration of 100 ​μg/mL ​(Fig. 4a—iii). GG and LAP-GG displayed rapid absorption kinetic with peak after 1 ​h and plateau at up to 24 ​h. Release of BSA after the addition of collagenase solution (Fig. 4a—iv) showed the greatest localisation capacity of LAP-GG compared with GG. LAP-free GG digestion with collagenase solution produced a linear release, while LAP-GG was found to release a negligible amount of BSA with a similar kinetic over 24 ​h.

### 3.5 CAM assay of VEGF-absorbed LAP-GG printed constructs

To test the potential to load drugs solubilised in the agarose gel bed while printing GG or LAP-GG, a bright dye (red and blue, respectively) was used to colour the agarose gel and absorption of the dye was investigated after printing and crosslinking of the scaffold (Fig. 4b). GG (Fig. 4b—i) and LAP-GG (Fig. 4b—ii) were printed in a red and blue dyed agarose gels, respectively. Image analysis indicated that LAP-GG strands were extensively loaded throughout the construct when compared with GG constructs. Loading of VEGF solubilised in agarose onto printed scaffolds was performed during extrusion of bulk cylindrical GG and LAP-GG bioink in the fluid gel. 3D constructs loaded with VEGF or HBSS (control) were implanted in the chick CAM model to evaluate angiogenesis and integration of the scaffolds with the CAM (Fig. 4c). GG, LAP-GG, GG-VEGF and LAP-GG-VEGF scaffolds were implanted on the CAM of the developing chick.

GG constructs were observed to integrate with the CAM with angiogenesis evident. Scaffold transparency allowed evaluation of penetrating vessels (small in number) through the structure with membrane vessels directed towards the sample. LAP-GG samples were also observed to integrate with the CAM. Blood vessels were found to be present around the outer surface of the construct (arrows) with blood vessels converged towards the integrated sample (arrows). VEGF absorbed on GG produced no significant effects compared with VEGF-free GG, although the sample was integrated with vessels around the sample (arrows) and minor capillaries around the construct (arrow). LAP-GG samples absorbed with VEGF resulted in integration after 7 days of incubation. Vessels were observed around the construct, with major vessels converging towards the construct. Chalkley scores (Fig. 4d) confirmed integration and vessel penetration through GG constructs. LAP-GG and VEGF-absorbed GG constructs showed no significant differences in membrane integration. LAP-GG-VEGF were found to significantly integrate with the CAM compared with the GG control (*p*<0.0001), LAP-GG and GG-VEGF (*p*<0.01).

## 4 Discussion

Recent advances in bioprinting technology have shown the potential of using a secondary gel to print a soft bioink to provide a support before post-printing crosslinking [29]. In this study, LAP nanoclay has been used to aid the printability and functionality of a common biomaterial (GG) printed in an agarose fluid gel bed. The inclusion and printing of C2C12 ​cells provided seminal data on the functionality of LAP-GG scaffolds. Drugs such as VEGF could be loaded in the agarose gel promoting LAP-GG absorption during printing supporting vascular infiltration in CAM model.

LAP nanoclay can aid printability [24], shape fidelity [30], mechanical properties [25], cell viability and drug delivery [12]. Jin et al. [31] recently reported on the use of an LAP gel formulation as a support bed for printing soft alginate--gelatin material. Bioink composition, viscoelastic properties, printing parameters and LAP concentration positively influenced printability and construct shape fidelity [4]. Bioink deposition was highly dependent on the concentration of the LAP bed (2–4% w/v) resulting in printed filaments with well-defined shapes and limited swelling; however, failed to specify an optimal printability window or functional properties. The study authors have used LAP in combination with GG ​to augment free-form printing functionality. GG is a low-cost, non—animal-derived polysaccharide widely reported in the tissue engineering literature for its biocompatible and drug-delivery properties [16]. GG is thermoresponsive and polymeric chains can crosslink in a double-helix after a decrease in the temperature in a thermoreversible transition [17]. However, GG has been combined with several other polymers or fillers (e.g. alginate [32], GelMA [[33], [34], [35]], carbon nanotubes [19]) to generate multifunctional hydrogel platforms for printing applications [36].

Gel stability is profoundly influenced by pH and ionic strength of the submerging medium [23]. Therefore, the effect of LAP addition on GG swelling ability, sol fraction and mass loss were investigated. LAP and GG content percentages were maintained at low concentrations with a view to examine changes in the physical parameters according to the serial increase of nanoclay content. PBS and HBSS were used as solutions containing a different ionic content. The sol fraction was found not to display any significant differences between LAP-GG formulations and controls. Nevertheless, L0.5G1, L1G1, G2 and L1G2 showed a significant increase in sol fraction percentage when immersed in PBS compared with HBSS solutions. These results suggest the possibility of PBS to induce greater swelling, possibly forcing the release of Ca ions from crosslinked LAP-GG because of the difference in molarity [37]. Mass swelling ratio was influenced by the ionic content of the PBS and HBSS solutions. Interestingly, LAP-GG formulations conditioned by the incubation in PBS induced the swelling of cast gels. In contrast, calcium-rich HBSS conditioned scaffolds were limited in their swelling behaviour. The results of this study are in agreement with Pacelli et al. [23], who reported the enhanced swelling of LAP-GG in deionised water compared with a solution of sodium chloride (NaCl). Possibly, the positive Na^+^ ions can contrast the negative surface of LAP nanoparticles that otherwise would interact strongly with the rest of the LAP negative charge inducing a repulsive action. Grasdalen et al. [14] studied the modulus of rigidity (N/cm^2^) of GG material in the presence of different ions. CaCl_2_ was found to influence GG positively compared with a solution without any salt, limiting the swelling range suggesting a tighter polymeric network.

Investigation of the swelling properties of LAP-GG and GG showed that the addition of only 1% (w/v) of nanosilicate could regulate the swelling behaviour of the hydrogel ink, as initially confirmed by the sol fraction, mass loss investigation and as the authors have previously reported [23]. LAP-GG displayed reduced swelling compared with GG when printed in air. Critically, LAP-GG showed significantly lower swelling than GG when printed in agarose confirming the hypothesis that LAP nanoparticles interact closely with gellan polymeric chains in the gel matrix. These results highlight the potential of nanoclay to improve print fidelity in a fluid gel medium and enhance bioink mechanical properties. Increased pressure was required to extrude the nanocomposite gel compared with GG to achieve similar shear resulting in greater printing fidelity after deposition [38]. The storage modulus decrease was noted after 10% shear, showing a limited printability window.

A more porous structure was observed in LAP-GG constructs in comparison with the silicate-free GG control, previously reported to show limited internal porosity [16,39]. Silicate-free GG was denser with a lower degree of internal porosity. Larger internal pores were evident in LAP-GG 3D constructs, suggesting a repulsive action of LAP nanodiscs inclusion in GG network as confirmed by the mass swelling ratio results. Printing of acellular bioinks is generally influenced by the swelling behaviour of the gels before and after crosslinking in air and in the supporting bath [7,29]. LAP inclusion allowed for greater resolution during deposition both in air and in agarose.

C2C12 evaluation on LAP-GG demonstrated sustained viability and improved function after 7 days of culture compared with the GG control. Cell proliferation of cells printed in GG appeared impaired in comparison with LAP-GG cell-laden constructs. Cells encapsulated in the soft hydrogels can potentially escape and proliferate on the tissue culture plastic as previously described [40]. In contrast, nanocomposite LAP-GG with enhanced mechanical properties to GG ​show higher cell retention capacity because of the stiffer matrix retaining cells in the scaffold network. High cell retention and viability provide evidence for the functional potential of LAP-GG.

In osteogenic media, cells were found to express significantly greater ALP staining. Particularly, osteogenic conditioning of cell-laden LAP-GG scaffolds were found to be stained intensely for ALP. Douglas et al. [16] recently reported the use of an ALP-GG hydrogel mineralisation protocol *in vitro.* ALP enzyme inclusion in GG network resulted in mineralisation when incubated in media conditioned with calcium phosphate (CaP) and magnesium glycerolphosphate (MgGP). GG gels were found to be extensively mineralised depending on Ca and Mg concentration in solution. Similarly, both GG and LAP-GG were crosslinked with 1M CaCl_2_. Residual Ca ions entrapped in the gel network ​can enhance mineralisation. However, GG hydrogels appeared to show greater functionality in basal and osteogenic media when LAP nanoclay containing a small percentage of Mg ions was included in the blend. This is consistent with previous data demonstrating the ability of LAP to enhance osteogenic differentiation, particularly associated with upregulated ALP production and matrix mineralisation (CaP) [41].

LAP has been shown, alone [11] or when blended with polymeric materials (e.g. alginate-methylcellulose [12], GelMA [13], PEG-PLA [42], pH-sensitive poly(N-vinylpyrrolidone) (PVP) [43]), to aid the localisation of drugs of interest. Compounds can be retained *in situ* to better functionally stimulate the local microenvironment. Lysozyme and BSA were used as BMP-2 and VEGF analogues drugs. Further investigations will be needed to confirm BMP-2 bioactivity when localised in LAP-GG novel bioink formulations. However, the current results show a promising localisation/release kinetic useful in aiding bone regeneration compared with silicate-free GG [44]. Lysozyme and BSA absorption/release kinetics showed the possibility to rapidly load a greater amount of compound in LAP-GG and a lower sustained release over time compared with the GG control. This property was harnessed in combination with 3D deposition technology, to load and produce scaffolds for angiogenesis. Following a previous study [45] where compounds of interest (ibuprofen, acetaminophen, indomethacin) were studied to diffuse through agarose with similar diffusion coefficient as in water, dyes in agarose fluid gel were found to penetrate LAP-GG with a greater depth than GG-printed constructs confirming greater absorption capacity of nanoclay bioink. The CAM assay [46] was used to investigate integration of the 3D-printed scaffolds with the vascularised membrane of the developing chick. Cylindrical bulk scaffolds were printed, and loaded while printing with VEGF solution in the agarose fluid gel. A fluid gel bed was used for the first time as a functional loading platform, working as a support and drug reservoir during printing. This offers the practical advantage of printing and loading simultaneously, providing a single-step fabrication platform for clinical skeletal application. As previously demonstrated [45], drugs result to diffuse similarly when suspended or solubilised in agarose and in aqueous environment. Therefore, LAP-GG printing in drug-loaded agarose bath results particularly attractive as a single-step clinically relevant implant production by printing living cells while loading drugs simultaneously.

The CAM assay demonstrated the efficacy of VEGF-loaded LAP-GG constructs compared with controls with enhanced angiogenesis. Absorption and localisation of VEGF mediated by LAP has been previously reported [11] confirming the ability of LAP gels to stimulate and guide angiogenesis both *in vitro* and *in vivo* after a brief exposure to VEGF solution. Release of VEGF has been investigated exclusively from GG-alginate bioinks [39] and resulted in similar kinetics as BSA release from silicate-free GG *in vitro*. *Ex vivo* results showed a greater effect of LAP-GG+VEGF suggesting greater ability to localise VEGF and providing a chemotactic platform beneficial for vascular infiltration. Further studies will seek to investigate the ability of clay-based bioinks to produce implantable scaffolds that can be functionalised during printing, providing a one-step procedure for the fabrication of clinically relevant constructs.

## 5 Conclusions

Free-form printing offers a promising biofabrication technology. The present study demonstrates that LAP-GG nanocomposite bioink offers significant potential in printing fidelity, with tailorable swelling characteristics and enhanced shape retention compared with silicate-free GG. LAP inclusion increased cell-laden bioink functionality, as evidenced by elevated ALP expression after 1 day of culture. Absorption/release properties of the LAP-GG blend were dependent on clay incorporation in the GG matrix. Functionality of cell and VEGF absorbed scaffolds, determined using the CAM assay, demonstrated integration with the CAM and significantly enhanced angiogenesis. In summary, the authors have demonstrated the unique potential of a novel LAP-GG bioink printed in agarose fluid gel, to produce a versatile system able to produce large-scale functional constructs offering promising applications for tissue skeletal reparation.

## Conflicts of interest

The authors declare that they have no known competing financial interests or personal relationships that could have appeared to influence the work reported in this paper.
